# Optogenetic and pharmacological interventions link hypocretin neurons to impulsivity in mice

**DOI:** 10.1038/s42003-023-04409-w

**Published:** 2023-01-19

**Authors:** Susan M. Tyree, Kimberly J. Jennings, Oscar C. Gonzalez, Shi-bin Li, Janet R. Nicholson, Moritz von Heimendahl, Luis de Lecea

**Affiliations:** 1 Department of Psychiatry and Behavioral Sciences, Stanford School of Medicine, Stanford, CA USA; 2 Boehringer Ingelheim Pharma GmbH & Co.KG, Biberach, Germany; 3 Present Address: Atlantia Clinical Trials, Cork, Ireland; 4 Present Address: University of Texas, Austin, TX USA

**Keywords:** Motivation, Neural circuits

## Abstract

Neurons in the lateral hypothalamus expressing the neuropeptide Hypocretin, also known as orexin, are known critical modulators of arousal stability. However, their role in the different components of the arousal construct such as attention and decision making is poorly understood. Here we study Hypocretin neuronal circuit dynamics during stop action impulsivity in a Go/NoGo task in mice. We show that Hypocretin neuronal activity correlates with anticipation of reward. We then assessed the causal role of Hypocretin neuronal activity using optogenetics in a Go/NoGo task. We show that stimulation of Hypocretin neurons during the cue period dramatically increases the number of premature responses. These effects are mimicked by amphetamine, reduced by atomoxetine, a norepinephrine uptake inhibitor, and blocked by a Hypocretin receptor 1 selective antagonist. We conclude that Hypocretin neurons have a key role in the integration of salient stimuli during wakefulness to produce appropriate and timely responses to rewarding and aversive cues.

## Abstract (web summary)

Optogenetic or pharmacological modulation of hypocretin neurons impacts mouse impulsivity in a Go/No-Go task, suggesting that these neurons play a key role in integrating salient stimuli and guiding responses to various environmental cues.

## Introduction

The Hypocretins (Hcrts), also known as orexins, are two neuropeptides derived from the same precursor^1,2^. Neurons that produce Hcrt peptides are restricted to the lateral hypothalamic area, but their projections extend broadly throughout the brain^3^. Previous studies have shown that integrity of the Hcrt system is essential for arousal stability; loss of Hcrt neurons in dogs, mice and humans results in narcolepsy with cataplexy. This stability is thought to be exerted by integrating multiple variables from local hypothalamic connections as well as afferents from hippocampus, septum and amygdala^4^.

In addition to the demonstrated role in arousal state transitions, multiple lines of evidence have placed the hypocretin/orexin system as an important relay in the processing of brain reward^5,6^. We and others showed that Hcrt R antagonism reduces motivation to seek a reward^7^, and blocks stress reinstatement of cocaine seeking^8,9^. This effect is likely due to a long-lasting increase in dopaminergic excitability elicited by Hcrt release^10–12^ through HcrtR1 signaling^13,14^.

Impulsivity, often defined as action without forethought or regard for consequences, is an essential feature of numerous psychiatric conditions including addiction and bipolar disorder^15,16^. An important common feature of arousal and addiction resides in the integration of salient signals to make appropriate goal-oriented decisions. We previously showed that activity of Hcrt neurons correlates with exposure to stimuli of both positive and negative valence^17,18^. However, whether Hcrt activity elicited by those stimuli has any effect on decision making is unknown. Here we have studied the role of Hcrt activity in decision making and action impulsivity by modulating the Hcrt system using pharmacology and optogenetics during an established Go/NoGo task.

## Results

### Hcrt neuronal activity correlates with stimulus salience

**Fig. 1:**
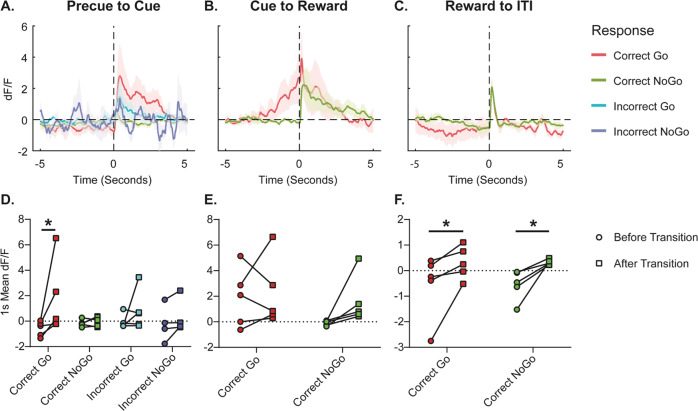
Hcrt Fiber Photometry During Go/NoGo Performance. Mean ΔF/F from Hcrt neurons 5 s before and 5 s after (**A**, **D**) Precue to Cue, (**B**, **E**) Cue to Reward, or (**C**, **F**) Reward to ITI transitions in the Go/NoGo task. **A**–**C** Time 0 and vertical dotted line denote the transition point. Color hue denotes animals’ response in the Go/NoGo task. Shaded region represents S.E.M. Bottom row (**D**–**F**) displays mean signal during the 1 s before and 1 s after the transition labeled above. *denotes difference between groups (Bonferroni’s test, *p* < 0.05).

We used fiber photometry to monitor the activity of Hcrt neurons in a Go/NoGo task. We trained Hcrt-IRES-cre knockin mice^18^ on the Go/NoGo task up to 70% accuracy, infused a viral vector encoding GCamp6f and implanted an optical fiber in the lateral hypothalamus (Supplementary Fig. 3). We recorded Hcrt neuronal activity throughout the Go/NoGo task and offline analyzed signal change during transitions between task phases (Precue, Go and NoGo Cues, Reward, ITI). As shown in Fig. 1A and D, calcium responses tended to increase at the transition from precue to cue periods, particularly in animals that responded correctly to the Go cue (Time x Transition Interaction F(1,4) = 2.69, *p* = 0.10). Correct Go traces were significantly different from Precue (Fig. 1D; *p* = 0.03). This signal is in contrast with the low levels of activity observed during the NoGo Cue period (Fig. 1B). Animals that had incorrect responses showed moderate, but significant differences in calcium signals upon cue exposure, consistent with a response to salient stimuli^18^. **Calcium** signals progressively increased during the Go Cue period and reached peak levels coincidental with delivery of a reward (Fig. 1B) (Time F(1,4) = 9.27, *p* = 0.04). In contrast, the calcium activity profile of Hcrt neurons remained low during the NoGo cue, but also showed a peak immediately after nosepoke. The transition from reward to the end of the trial into the inter-trial-interval period also showed a peak in activity (Fig. 1C, F) (Time F(1,4) = 7.88, *p* = 0.048), but both Correct Go and NoGo groups showed similar responses (Time x Transition F(1,4) = 0.007, *p* = 0.94). No fluorescent signal was detected in wild-type (Hcrt-IRES- cre -) mice (Supplementary Fig. 1).

### Optogenetic stimulation of Hcrt Neurons during Go/NoGo task

**Fig. 2:**
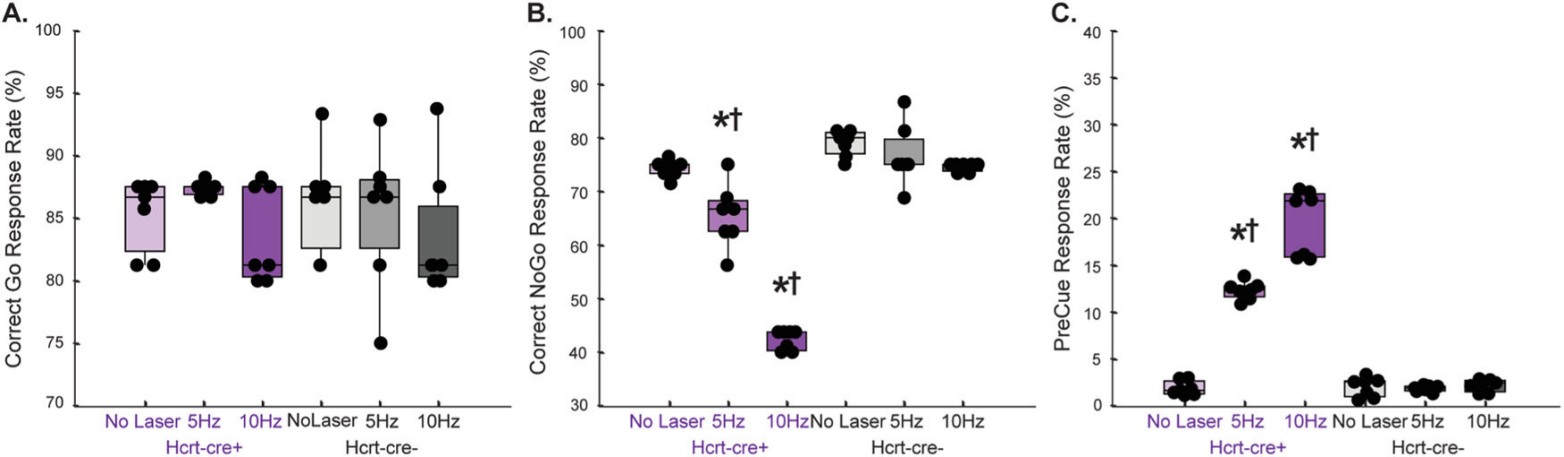
Stimulating Hcrt neurons increases impulsivity. The effects of 5 or 10 Hz optogenetic stimulation of Hcrt neurons on Go/NoGo Behavior. **A** Hcrt stimulation has no effect on probability of a correct Go response but does (**B**) reduce probability of a correct NoGo response and (**C**) increase precue response rate. Effects are not seen in Hcrt-cre- controls (gray). Color indicates genotype and shade indicates laser frequency. *denotes difference from No-Laser control trials within the same genotype (Bonferroni’s test, *p* < 0.05) and † denotes difference from Hcrt-cre- controls at that particular laser frequency. Box plots indicate averages and standard deviation. Whiskers span maxima and minima values.

To determine whether the peak of Hcrt activity was causal to impulsive action, we used optogenetic stimulation of Hcrt neurons during the first 5 s of the Go and NoGo cues. We chose parameters that are consistent with in vivo recordings of Hcrt neurons^19^ at 5 and 10 Hz. Stimulation during the Go cue did not significantly affect the number of responses (*P* > 0.05) (Fig. 2A). However, Hcrt stimulation during the NoGo cue dramatically reduced the probability of correct NoGo trials (*p* < 0.001 RM-ANOVA with Bonferroni multiple comparisons) (Fig. 2B; Supplementary Movies 1 and 2). Interestingly, optogenetic stimulation of Hcrt during the pre-cue period increased premature responses as well in Hcrt-cre animals but not in wild-type control mice (*P* > 0.05, RM-ANOVA) (Fig. 2C). These results strongly suggest that Hcrt neurons respond to salient signals associated with a reward, and activity is suppressed if behavioral inhibition is required.

### Pharmacological manipulation of Go/NoGo performance

Hcrt exerts its action on two GPCRS that bind differentially to Hcrt1 and Hcrt2. Studies in knockout animals indicate that both HcrtR1 and HcrtR2 signaling affect sleep/wake stability^20^, whereas HcrtR1 modulates the effects on brain reward function^21^. We therefore used a selective HcrtR1 antagonist to test whether HcrtR1 signaling is necessary for Hcrt’s effect on impulsivity.

**Fig. 3:**
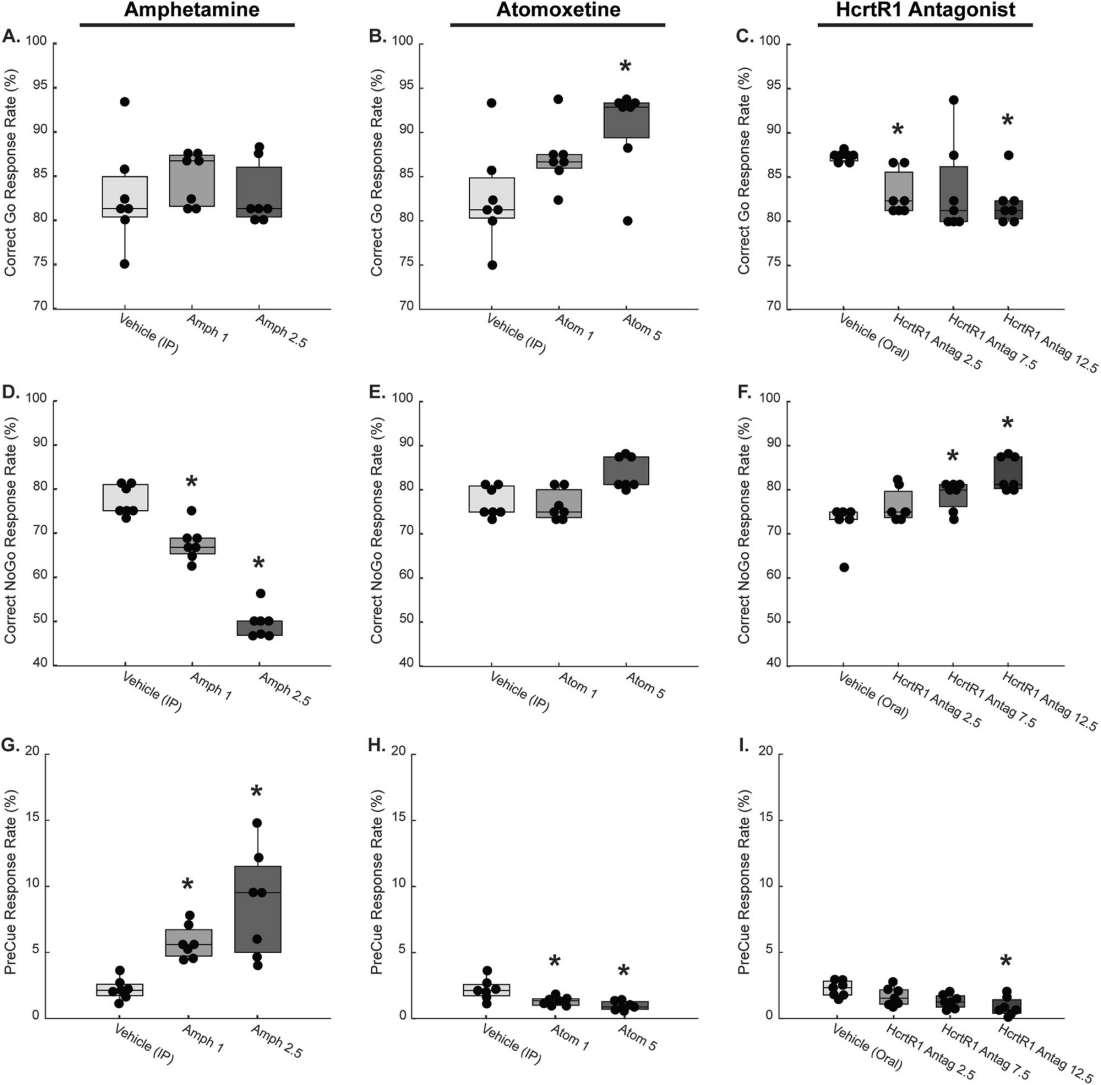
Pharmacological Manipulation of Go/NoGo Performance. Amphetamine decreases correct NoGo response rate (**D**) and increases PreCue response rate (**G**), but does not alter correct Go response rate (**A**). In contrast, atomoxetine increases correct Go response rate (**B**) and decreases PreCue response rate (**H**), but does not alter correct NoGo response rate (**E**). Selective HcrtR1 antagonism decreases correct Go response rate (**C**) and PreCue response rate (**I**) and increases correct NoGo response rate (**F**). Vehicle data are shared between amphetamine and atomoxetine groups (delivered I.P.), whereas HcrtR1 antagonist data are compared to vehicle delivered in the same modality (oral gavage). *denotes difference between from vehicle treatment (Bonferroni’s test, *p* < 0.05). Box plots indicate averages and standard deviation. Whiskers span maxima and minima values.

To calibrate the effect of HcrtR1 antagonists, we evaluated the animals’ responses on the Go/NoGo task following injections of different doses of Amphetamine (Figs. 3A, D, G), (1 and 2.5 mg delivered 10 min before the assay, a psychostimulant known to increase impulsive choice^22^ or Atomoxetine (Fig. 3B, E, H), a noradrenergic reuptake inhibitor that improves performance in the Go/NoGo task^23^. Indeed, treatment with amphetamine, dose dependently reduced the NoGo probability (*P* < 0.05 RM-ANOVA) (Fig. 3D), whereas atomoxetine slightly improved the mice performance (*P* < 0.05 RM-ANOVA) (Fig. 3B, H). Similarly to atomoxetine, the selective HcrtR1 antagonist dose-dependently increased the No Go probability and reduced the Pre-Cue response rate (*P* < 0.05 RM-ANOVA) (Fig. 3F, I).

### Pharmacology of Hcrt-induced premature responses

**Fig. 4:**
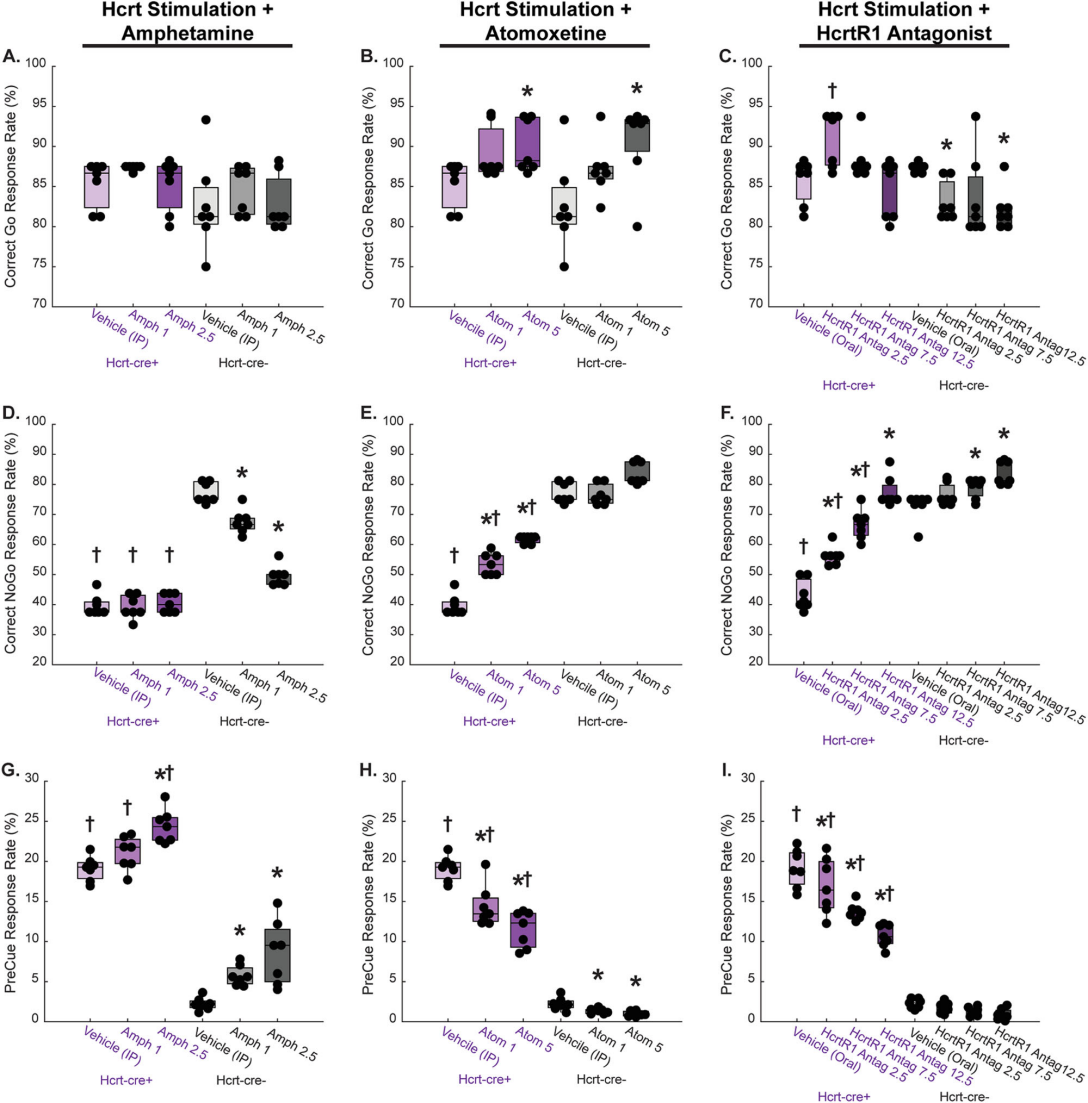
Pharmacology of Hcrt-induced premature responses. The effects of 10 Hz Hcrt stimulation concurrent with administration of amphetamine (**A**, **D**, **G**), atomoxetine (**B**, **E**, **H**), or HcrtR1 antagonist (**C**, **F**, **I**) at various dosages on correct Go response rate (**A**, **B**, **C**, stimulation delivered during the first 5 s of the Go Cue Period), correct NoGo response rate (**D**, **E**, **F**, stimulation delivered during the first 5 s of the NoGo Cue Period), and PreCue response rate (**G**, **H**, **I**, stimulation delivered during the last 5 s of the PreCue Period) are shown. Color indicates genotype (purple for hcre-cre+ and gray for controls) and shade indicates dosage (darker for higher dosages). Vehicle data are shared between amphetamine and atomoxetine groups (delivered I.P.), whereas HcrtR1 antagonist data are compared to vehicle delivered in the same modality (oral gavage). *denotes difference from vehicle within same genotype (Bonferroni’s test, *p* < 0.05); † denotes difference from Hcrt-cre- controls at that particular dosage (Bonferroni’s test, *p* < 0.05).

We then compared the operant responses in a Go/NoGo paradigm following optogenetic Hcrt stimulation and systemic pharmacological treatment with amphetamine (Fig. 4A, D, G), atomoxetine (Fig. 4B, E, H), or a selective HcrtR1 antagonist (Fig. 4C, F, I). The probability of premature responses during the preCue period was increased by systemic administration of Amphetamine, similarly to Hcrt neuronal stimulation (Fig. 4G) (*P* < 0.05 main effects of treatment, genotype in two-way RM-ANOVA). In contrast, treatment with atomoxetine, a noradrenergic uptake inhibitor known to increase attention and decrease impulsivity^24^, partially recovered the effects of Hcrt photostimulation (Fig. 4E, H) (*P* < 0.05 main effects and interaction of treatment and genotype in two-way RM-ANOVA). The HcrtR1 antagonist fully recovered the increase of premature responses elicited by Hcrt stimulation (Fig. 4F) (*P* < 0.05 main effects and interaction of treatment and genotype in two-way RM-ANOVA).

## Discussion

Impulsivity, which leads to impaired decision making, is associated with many psychiatric and behavioral disorders, such as attention-deficit/hyperactivity disorder, eating and substance abuse disorders^25–27^. Multiple studies have established a relationship between caffeine consumption, sleep loss, risk behaviors, and impulsivity^28,29^, although the neuronal mechanisms of such relationships are unknown. The Yerkes-Dodson Law states that there is a relationship between physiological arousal and performance up to a certain point, where an excess of arousal leads to poor performance. Indeed, evidence exists that hypoarousal in Hcrt-deficient narcoleptic patients results in attention deficits^30^. Here we used a Go/NoGo task to evaluate performance and motoric impulsivity behavior in mice and tested whether arousal driven by the neuropeptide Hcrt followed the Yerkes-Dodson Law. We show that hyperarousal induced by optogenetic stimulation of Hcrt neurons at frequencies consistent with their phasic activity profile^31,32^ also decreases attention performance.

Increased impulsivity caused by Hcrt activity is consistent with other reports showing decreased impulsivity for cocaine upon treatment with suvorexant, a dual HcrtR antagonist^33^. Hcrt neurons appear to be activated by a plethora of salient stimuli of both positive and negative valences^17,18^. Thus, Hcrt activity may be interpreted as an alert/emergency signal that quickly engages monoaminergic arousal circuits. Using cFos, Freeman et al. ^34^ showed activation of medial, but not the lateral hypothalamic Hcrt neurons correlated with greater accuracy on the Go/NoGo task. The poor temporal resolution of cFos immunoreactivity (60 min after the Go/NoGo session) prevents direct comparisons with our studies. One limitation of our results is that exact cannula placement could not be verified anatomically due to poor tissue quality. We did, however, test accurate placement before the optogenetic experiments by monitoring sleep/wake transitions following 10 Hz stimulation. This method proved extremely reliable in our previous work^17^. We also verified accuracy of injection conditions and eutopic expression of the transgenes in new animals (Supplementary Fig. 2 and 3).

Which mechanism drives impulsivity elicited by Hcrt neurons? The neural circuitry underlying Go/NoGo has been associated with both the functional and structural integrity of brain systems known to be compromised in stimulant drug dependence in humans^35,36^ and rodents^26,27^. The ability to inhibit actions after a habit has formed has classically been linked to neuronal loops between ventral tegmental area (VTA) and nucleus accumbens (NAcc) shell as well as corticostriatal loops. However, how these structures are modulated by arousal and attention is still poorly understood. Interactions between NAcc shell and the VTA have been proposed as the main substrate of waiting impulsivity, whereby VTA neurons project to GABAergic cells in the NAcc shell, which project back to GABA interneurons in the VTA. Impulsive premature responding is associated with decreased dopamine release in the core and increased dopamine release in the shell subregion^37^. Substantial evidence shows reciprocal connections between Hcrt, D2 receptor-containing neurons in the NAcc shell and the VTA. Hcrt1 increases firing of lateral and medial NAcc shell neurons^38^. Recently Gonzalez et al. ^39^, showed that Hcrt neurons increased activity during approach to food, and this activity declined to baseline at the start of consummatory behavior, mediated by reciprocal interactions between the Shell of the NAcc and Hcrt neurons. The reduced correct Go responses observed after treatment of a Hcrt R1 antagonist (Fig. 2C) are unlikely due to increased sleepiness^20^, since Hcrt R1 knockout mice have a mild sleep phenotype; instead these mice indicate that HcrtR1 is necessary for food-reinforced responding, motivation, or both^21^. Blomeley et al. ^40^ described a direct Hcrt→D2 excitatory circuit and showed that D2 cell activity is necessary for Hcrt-dependent innate risk-avoidance in mice^40^. Dynorphin, which is co-released by Hcrt+ in the LH, inhibits the majority of medial NAcc shell- and basolateral amygdala-projecting dopamine neurons but reduces firing only in a small fraction of those that project to the lateral NAcc shell. Activation of Kappa opioid receptors have been suggested to increase impulsivity in the 5CSRRT^41^. Our optogenetic stimulation of Hcrt neurons may have increased Dynorphin release^42,43^. Thus, it is conceivable that changes in the E/I ratio elicited by Hcrt release in the NAcc shell could elicit premature responses either by binding to Hcrt receptors on D2 neurons or NPY + interneurons. It is noteworthy that the neuromodulatory effect of Hcrt in the NAcc seems to be specific, as chemogenetic activation of VTA neurons does not affect impulsivity^44,45^. Hcrt activity may also shift the E/I balance of hypothalamic outputs: the LH(GAD65) neuron excitation induces elevated locomotor activity, while inhibition of LH(GAD65) neuron natural activity depresses voluntary locomotion^46^. The Hcrt → LH(GAD65) circuit may therefore assist in creating the drive to run, reflected in the Go/NoGo assay as increased premature responses.

In addition to the canonical VTA→ NAcc shell circuit, optogenetic Hcrt stimulation activates the locus coeruleus (LC)^47^, a structure also involved in impulsive behavior mediated by noradrenaline release in the prefrontal cortex (PFC)^48^ or the NAcc^49^. Norepinephrine (NE) release in the NAcc shell plays an important role in the effects of atomoxetine on impulsivity, whereas NE in the PFC attenuates the effects of amphetamine on impulse behavior. Since Hcrt optogenetic stimulation did not change the effects of amphetamine on impulsivity, NE appears to modulate Hcrt action in the NAcc.

Using fiber photometry, we have shown that Hcrt neuronal activity in the lateral hypothalamus peaks at the time of delivering a reward in Go trials, consistent with the reported 74% of neurons increasing their firing rate in global recordings of a choice task^50^. Recent studies by Burdakov et al. ^46^ and our own laboratory^17^ indicate that Hcrt neurons are sensitive to multiple salient stimuli and the increased Ca^2+^ concentration during the Go session may reflect such salience (Fig. 1). Similar photometry profiles were observed when recording responses of TH + noradrenergic neurons in the Locus coeruleus, a brain structure critically involved in attention. Previous work from our laboratory showed that Hcrt neurons densely project to LC and that optogenetically blocking LC neuronal activity prevents Hcrt-induced sleep to wake transitions^47^. The photometry profiles of Hcrt and LC neurons were also similar when recorded during correct NoGo trials and during premature responses in NoGo sessions. The HcrtLC connection is likely signaled through HcrtR1 receptors based on their reported expression pattern in the LC^51,52^. Accordingly, HcrtR1 antagonists were able to reduce the premature responses elicited by amphetamine.

ADHD is characterized by a developmentally inappropriate level of inattentiveness, impulsivity and/or hyperactivity, and atomoxetine and other stimulants have been used to treat adults with this disorder^53^. Indeed, here we show that atomoxetine, a noradrenergic uptake inhibitor, partially restores normal responses following impulsivity elicited by optogenetic Hcrt stimulation. A selective Hcrt R1 antagonist appeared more efficacious at rescuing the probability of inhibitory behavior in the impulsivity test, suggesting a possible clinical application in the treatment of psychiatric disorders with maladaptive impulsivity.

Here we have demonstrated a causal relationship between activation of Hcrt neurons, and both waiting and stopping impulsivity. Optogenetic stimulation of Hcrt neurons increased premature responses in NoGo trials, whereas a HcrtR1 selective antagonist reduced the effect of amphetamine on a Go/NoGo task. This effect is likely mediated through dopaminergic and noradrenergic mechanisms in the striatum and PFC. The robustness and specificity of HcrtR1 antagonists on this task makes them excellent pharmacological tools to treat ADHD and other disorders associated with impulsivity.

## Methods

### Ethics statement

All experiments were carried out in accordance with the US National Institutes of Health Guide for the Care and Use of Laboratory Animals guidelines and were approved by the Stanford University Administrative Panel on Laboratory Animal Care (protocol ID #18787).

### Drug treatment

Animals received each drug/dose in a random order to protect against order effects, with at least 3 days between treatment days to allow for sufficient washout. Atomoxetine hydrochloride was obtained from Sigma (Y0001586) and was dissolved in 0.9% NaCl (saline) which was administered via intraperitoneal injection at a dose of either 5 mg/kg or 10 mg/kg 30 min prior to the start of the Go/NoGo test. D-amphetamine hemisulfate was obtained from Sigma (A5880 and was dissolved in 0.9% NaCl (saline) which was administered via intraperitoneal injection at a dose of either 1 mg/kg or 2.5 mg/kg 10 min prior to the start of the Go/NoGo test^22,54^). Additionally, a HcrtR1 antagonist was obtained from Boehringer Ingelheim (patent WO2017/178339) and was dissolved in 0.5% hydroxyethylcellulose (Sigma, 525944) and 0.015% Tween 80 (Sigma, P1754) in water which was administered via oral gavage at a dose of either 2.5 mg/kg, 7.5 mg/kg, or 12.5 mg/kg. In addition to the 7 drug/dose groups, two control groups were included in which mice received either an intraperitoneal injection of saline 10 min prior to the start of the Go/NoGo test or received the vehicle solution used to administer the HcrtR1 compound via oral gavage 60 min prior to the start of the Go/NoGo test.

### Animals

Male Hcrt-IRES-Cre knock-in heterozygote mice (Hcrt-cre +) backcrossed onto C57BL6J background (N9) were bred in house, with wild type littermates (Hcrt-cre-) used as controls. The mice were housed in groups of up to five mice in plexiglass chambers with stable temperature (22 ± 1 ˚C), humidity (40–60%), and lighting conditions (9:00 am–9:00 pm dark; 9:00 pm–9:00 am light). At the time of the beginning of the training the mice weighed ~27 g. During training mice were transitioned over to a water-restriction paradigm, in which they were given access to water for 2–4 h at the end of their active period. All training, recording, and manipulations occurred during the dark period.

### Surgery

Mice were anaesthetized with a mixture of Ketamine (100 mg/kg) and Xylazine (20 mg/kg) and mounted onto an animal stereotaxic frame (David Kopf Instruments) and received injections of 0.3 μl AAV-DJ-EF1α-DIO-hChR2(H134R)-eYFP virus (2.5 × 10^12^ genome copies per ml, Stanford Virus Core) to the right or left lateral hypothalamus (LH) (AP: − 1.35 mm, ML: ± 0.95 mm, DV: −5.15 mm) with a 5 μl Hamilton microsyringe. A glass fiber (200 μm in diameter, Doric Lenses, Franquet, Québec, Canada) was implanted with the tip right above the injection site for optogenetic stimulations. For fiber photometry, 0.3 μl AAV vectors carrying genes encoding GCaMP6f (AAV-DJ-EF1α-DIO-GCaMP6f, 1.1 × 10^13^ genome copies per ml, Stanford Virus Core) were delivered to the right or left LH (AP:−1.35 mm, ML: ± 0.95 mm, DV: −5.15 mm) with a 5 μl Hamilton micro-syringe, and a glass fiber (400 μm diameter, 0.48 NA, Doric Lenses) was implanted with the tip at the injection site for later GCaMP6f signal acquisition. Hcrt-cre+ and Hcrt-cre- (control) mice received identical viral treatment.

### Behavioral paradigms: Go/NoGo training

**Fig. 5:**
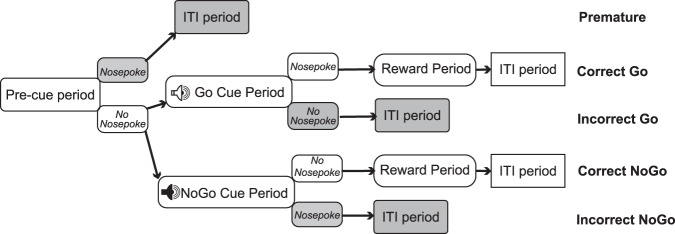
Schematic of the Go/NoGo task. After a variable length Precue period (duration 9–24 s, cage light on), Go and NoGo periods (duration 10 s or until nosepoke, cage light on) are signaled to the animal via a distinct auditory cue. ITI stands for inter-trial interval (duration 10 s, cage light off). Premature responses during the Pre-cue period and incorrect responses during the Cue period trigger progression to the ITI, as does conclusion of the reward period (duration 3 s, cage light on). Shadowed boxes indicate incorrect responses.

Animals were first trained to learn where reward delivery occurs by being trained on a Random Interval 60 s schedule until they were reliably investigating the nose-poke reward port (>200 nose-pokes per session) and reliably nose-poking during the reward period (until ~80% of reward periods showed at least one nose-poke). Following this, the mice were trained on the ‘Go Cue’ in a session of either 40 min or 60 trials (whichever came first) of only Go Cue trials. Once mice were reliably responding to the Go Cue (>70% accurate response to Go Cue across three consecutive training days) the ‘NoGo Cue’ was introduced so that the 40 min/60 trial session was a random distribution of 50% Go trials and 50% NoGo trials. Once mice were reliably responding accurately to both Go and NoGo cues (>70% correct responses to cues across three consecutive training days), the mice were considered ready for testing. Reliable accuracy was maintained between testing days with regular training (at least 5 days a week)—mice were only tested if their most recent training session showed >70% accuracy to both Go and NoGo cues (Fig. 5).

The following parameters were calculated per session: Hits: the number of times an animal produces a nose poke during the cue period of a Go trial in a particular session; False Alarms: the number of NoGo trials on which the animal produced a nose poke during the NoGo cue period; PreCue Response Rate: the total number of responses made during all the pre-cue periods and is divided by the total duration of all of the pre-cue periods. If no poke occurred during cue presentation the value is set to the maximum latency (maximum time of cue presentation).

### Neural activity recording/manipulation

For a fiber-photometry recording, Hcrt-cre+ and Hcrt-cre- mice were connected to a flexible recording cable and placed in the operant chamber. There, their GCaMP6f signal was recorded for 12 min, 1 min of baseline recording while the mouse was resting in the operant cage with no task running, and then 10 min of Go/NoGo trials (50:50 ratio of Go to NoGo trials), followed by an additional minute of post-session recording. Recordings were captured using equipment as described previously^55,56^. Briefly, 470 nm excitation light (M470F3, Thorlabs, NJ, USA) was sinusoidally modulated at 211 Hz using a custom Matlab program (MathWorks, Natick, MA, USA) and a multifunction data acquisition device (NI USB-6259, National Instruments, Austin, TX, USA). Excitation light passed through a GFP excitation filter (MF469-35, Thorlabs) and reflected by a dichroic mirror (MD498, Thorlabs) into a low-fluorescence patch cord (400 µm, 0.48 NA; Doric Lenses) via a fiber collimation package (F240FC-A, Thorlabs). The patch cord was connected to the animal’s implanted optic fiber via a zirconia sleeve (SLEEVE_ZR_2.50, Doric Lenses). GCaMP6f fluorescence was collected through the patch cord and passed through a GFP emission filter (MF525- 39, Thorlabs) and focused onto a photodetector (LA1540-A, Thorlabs; Model 2151, Newport, Irvine, CA, USA). The signal was then sent to a lock-in amplifier (30-ms time constant, Model SR830, Stanford Research Systems, Sunnyvale, CA, USA) synchronized to 211 Hz and then collected at 1 KHz with a custom Matlab script and a multifunction data acquisition device (National Instruments). GCaMP6f signals were aligned to the behaviors in the operant chamber via TTL pulses sent from the operant chamber and recorded in a parallel data stream via a custom Matlab script. To quantify the change of GCaMP6f signals, the values before state transitions were averaged as the baseline, and the area size (ΔF/F integral) between the baseline and GCaMP6f signal trace was determined and averaged for each individual mouse.

### Optogenetic stimulation of Hcrt neurons

To examine the effect of stimulating Hcrt neurons across the Go/NoGo task, Hcrt-cre+ and Hcrt-cre- mice were connected via a fiberoptic patch cord to a laser. The light intensity was calibrated to 10 mW at the tip with a light meter (Thorlabs). Then, fiber optic patch cord (MFP_200/240/900-0.22_3.0m_FC-MF2.5, Doric lenses) was connected to the glass fiber implant through a zirconia sleeve (SLEEVE_ZR_2.50, Doric lenses). To verify fiber placement, animals were confirmed to wake (initiate movement) within 20 s of stimulation (5 s at 5 Hz, 10 ms pulse width, 10 mW) delivered during sustained (30+ seconds) sleep-like behavior, as reported elsewhere^57,58^. Since tissue integrity was compromised in unfixed tissue, anatomical localization of the cannula was verified post mortem in a separate group of animals (see Supplementary Fig. 2). After habituation to the Go/NoGo apparatus with fiberoptic attachment, optogenetic stimulation with varying frequencies was performed at different points during the Go/NoGo trials (light intensity at the fiber tip: 10 mW, light pulse width: 15 ms; 5 and 10 Hz stimulation for 5 s were performed). The moment of laser stimulation was randomly distributed between 4 trial conditions: (1) no laser stimulation; (2) laser stimulation for the last 5 s of the PreCue Period; (3) laser stimulation for the first 5 s of the Go Cue Period; and (4) laser stimulation for the first 5 s of the NoGo Cue Period. Optogenetic stimulation parameters were selected based on previously reported validations of Hcrt optogenetic stimulation from our lab^18,56^.

### Immunocytochemistry

For colocalization of ChR2/GCaMP expression and Hypocretin immunoreactivity, we transcardially perfused mice with phosphate buffer followed by buffered 4% paraformaldehyde (pH7.4). Extracted brains were then postfixed in 4% paraformaldehyde for 2 h before being placed in 30% sucrose solution for cryoprotection for 48 h. Brains were then sectioned on a Leica cryostat at 30um and stored in phosphate buffer until staining. Slices were blocked in 5% Bovine Serum Albumin/0.5% Triton solution for 1 hr at 36 C. Slices were then incubated in primary antibody against hypocretin-A (Abcam ab6214) at a 1:250 dilution in 3% BSA/0.3% Triton solution overnight at 4 C. Slices were then washed in PBS followed by incubation with AlexaFlour594 at 1:1000 dilution in 3% BSA/0.3% TritonX100 solution at 36 C for 1.5-2 h. Sliced were then washed in PBS, and then mounted with DAPI.

### Statistics and reproducibility

All statistical analyses were completed using GraphPad Prism 8.4.1. Fiber photometry data were analyzed by Repeated Measures Analysis of Variance (RM-ANOVA) using transition type and time (before vs. after transition) as statistical factors. Because of unequal sample sizes across trial responses, fiber photometry data from the Precue-to-Cue transition were analyzed using a mixed-effects model. The effect of Hcrt optogenetic stimulation frequencies on behavior was assessed using RM-ANOVA with stimulation frequency and genotype as statistical factors. Similarly, the effect of pharmacological treatment on behavior was analyzed using RM-ANOVA with genotype and treatment as factors. Bonferroni’s multiple comparisons tests were performed post hoc to probe specific group differences (Supplementary Data 1). Data source for Figs. 2–4 can be found in Supplementary Data 2.

### Reporting summary

Further information on research design is available in the Nature Portfolio Reporting Summary linked to this article.

## Supplementary information

Supplementary Information

Description of Additional Supplementary Files

Supplementary Data 1

Supplementary Data 2

Supplementary Movie 1

Supplementary Movie 2

Reporting Summary

## Data Availability

The data sets generated and analysed during the current study are available in the Stanford Digital Repository [https://purl.stanford.edu/sf095mv6553. 10.25740/sf095mv6553].

## References

[1] de Lecea L (1998). The hypocretins: hypothalamus-specific peptides with neuroexcitatory activity. Proc. Natl Acad. Sci. USA.

[2] Sakurai T (1998). Orexins and orexin receptors: a family of hypothalamic neuropeptides and G protein-coupled receptors that regulate feeding behavior. Cell.

[3] Li SBin, Giardino WJ, de Lecea L (2017). Hypocretins and arousal. Curr. Top. Behav. Neurosci..

[4] Prober DA (2018). Discovery of hypocretin/orexin ushers in a new era of sleep research. Trends Neurosci..

[5] Koob GF, Volkow ND (2010). Neurocircuitry of addiction. Neuropsychopharmacology.

[6] Robinson TE, Berridge KC (2001). Incentive-sensitization and addiction. Addiction.

[7] Steiner N (2018). Hypocretin/orexin deficiency decreases cocaine abuse liability. Neuropharmacology.

[8] Boutrel B, Cannella N, de Lecea L (2010). The role of hypocretin in driving arousal and goal-oriented behaviors. Brain Res.

[9] Boutrel B (2005). Role for hypocretin in mediating stress-induced reinstatement of cocaine-seeking behavior. Proc. Natl Acad. Sci. USA.

[10] Baimel C, Borgland SL (2015). Orexin signaling in the VTA gates morphine-induced synaptic plasticity. J. Neurosci..

[11] Baimel C, Borgland SL (2012). Hypocretin modulation of drug-induced synaptic plasticity. Prog. Brain Res..

[12] España RA, Melchior JR, Roberts DCS, Jones SR (2011). Hypocretin 1/orexin A in the ventral tegmental area enhances dopamine responses to cocaine and promotes cocaine self-administration. Psychopharmacol. (Berl.).

[13] Perrey DA, Zhang Y (2020). Therapeutics development for addiction: orexin-1 receptor antagonists. Brain Res..

[14] Zhou L (2012). Orexin-1 receptor mediation of cocaine seeking in male and female rats. J. Pharmacol. Exp. Ther..

[15] Chamberlain SR, Sahakian BJ (2007). The neuropsychiatry of impulsivity. Curr. Opin. Psychiatry.

[16] Robbins TW, Gillan CM, Smith DG, de Wit S, Ersche KD (2012). Neurocognitive endophenotypes of impulsivity and compulsivity: towards dimensional psychiatry. Trends Cogn. Sci..

[17] Li SBin (2020). Hypothalamic circuitry underlying stress-induced insomnia and peripheral immunosuppression. Sci. Adv..

[18] Giardino WJ (2018). Parallel circuits from the bed nuclei of stria terminalis to the lateral hypothalamus drive opposing emotional states. Nat. Neurosci..

[19] Mileykovskiy BY, Kiyashchenko LI, Siegel JM (2005). Behavioral correlates of activity in identified hypocretin/orexin neurons. Neuron.

[20] Mieda, M. et al. Differential roles of orexin receptor-1 and -2 in the regulation of non-REM and REM sleep. *J. Neurosci*. 10.1523/JNEUROSCI.6506-10.2011 (2011). 10.1523/JNEUROSCI.6506-10.2011 PMC3732784 21525292

[21] Sharf, R. et al. Orexin signaling via the Orexin 1 receptor mediates operant responding for food reinforcement. *Biol. Psychiatry*10.1016/j.biopsych.2009.12.035 (2010). 10.1016/j.biopsych.2009.12.035 PMC2849869 20189166

[22] Loos M (2009). Activity and impulsive action are controlled by different genetic and environmental factors. Genes, Brain Behav..

[23] Pillidge K, Porter AJ, Vasili T, Heal DJ, Stanford SC (2014). Atomoxetine reduces hyperactive/impulsive behaviours in neurokinin-1 receptor “knockout” mice. Pharmacol. Biochem. Behav..

[24] Robinson ESJ (2008). Similar effects of the selective noradrenaline reuptake inhibitor atomoxetine on three distinct forms of impulsivity in the rat. Neuropsychopharmacology.

[25] Anderberg RH (2016). The stomach-derived hormone Ghrelin increases impulsive behavior. Neuropsychopharmacology.

[26] Dalley JW, Everitt BJ, Robbins TW (2011). Impulsivity, compulsivity, and top-down cognitive control. Neuron.

[27] Dalley JW, Robbins TW (2017). Fractionating impulsivity: neuropsychiatric implications. Nat. Rev. Neurosci..

[28] Roehrs T, Greenwald M, Roth T (2004). Risk-taking behavior: Effects of ethanol, caffeine, and basal sleepiness. Sleep.

[29] Heinz AJ, de Wit H, Lilje TC, Kassel JD (2013). The combined effects of alcohol, caffeine, and expectancies on subjective experience, impulsivity, and risk-taking. Exp. Clin. Psychopharmacol..

[30] Ramm, M. et al. The Perception and Attention Functions test battery as a measure of neurocognitive impairment in patients with suspected central disorders of hypersomnolence. *J. Sleep Res*. **27**, 273–280 (2018). 10.1111/jsr.12587 28771870

[31] Lee, M. G., Hassani, O. K. & Jones, B. E. Discharge of identified orexin/hypocretin neurons across the sleep-waking cycle. *J. Neurosci*. 10.1523/JNEUROSCI.1887-05.2005 (2005). 10.1523/JNEUROSCI.1887-05.2005 PMC6725432 16014733

[32] Karnani, M. M. et al. Rapid sensory integration in orexin neurons governs probability of future movements. *bioRxiv*10.1101/620096 (2019).

[33] Gentile TA (2018). Suvorexant, an orexin/hypocretin receptor antagonist, attenuates motivational and hedonic properties of cocaine. Addict. Biol..

[34] Freeman LR, Aston-Jones G (2020). Activation of medial hypothalamic orexin neurons during a Go/No-Go task. Brain Res.

[35] Tabibnia G (2011). Different forms of self-control share a neurocognitive substrate. J. Neurosci..

[36] Li CSR, Milivojevic V, Kemp K, Hong K, Sinha R (2006). Performance monitoring and stop signal inhibition in abstinent patients with cocaine dependence. Drug Alcohol Depend..

[37] Diergaarde L (2008). Impulsive choice and impulsive action predict vulnerability to distinct stages of nicotine seeking in rats. Biol. Psychiatry.

[38] Baimel C, Lau BK, Qiao M, Borgland SL (2017). Projection-target-defined effects of orexin and dynorphin on VTA dopamine neurons. Cell Rep..

[39] González JA (2016). Inhibitory interplay between orexin neurons and eating. Curr. Biol..

[40] Blomeley C, Garau C, Burdakov D (2018). Accumbal D2 cells orchestrate innate risk-avoidance according to orexin signals. Nat. Neurosci..

[41] Funk D, Tamadon S, Coen K, Fletcher PJ, Lê AD (2019). Kappa opioid receptors mediate yohimbine-induced increases in impulsivity in the 5-choice serial reaction time task. Behav. Brain Res..

[42] Matzeu A, Kallupi M, George O, Schweitzer P, Martin-Fardon R (2018). Dynorphin counteracts orexin in the paraventricular nucleus of the thalamus: cellular and behavioral evidence. Neuropsychopharmacology.

[43] Li Y, Van Den Pol AN (2006). Differential target-dependent actions of coexpressed inhibitory dynorphin and excitatory hypocretin/orexin neuropeptides. J. Neurosci..

[44] Boekhoudt L (2017). Chemogenetic activation of midbrain dopamine neurons affects attention, but not impulsivity, in the five-choice serial reaction time task in rats. Neuropsychopharmacology.

[45] Fitzpatrick CM (2019). Differential effects of chemogenetic inhibition of dopamine and norepinephrine neurons in the mouse 5-choice serial reaction time task. Prog. Neuro-Psychopharmacol. Biol. Psychiatry.

[46] Burdakov D (2020). How orexin signals bias action: hypothalamic and accumbal circuits. Brain Res.

[47] Carter ME (2012). Mechanism for hypocretin-mediated sleep-to-wake transitions. Proc. Natl Acad. Sci. USA.

[48] Bari A, Robbins TW (2013). Noradrenergic versus dopaminergic modulation of impulsivity, attention and monitoring behaviour in rats performing the stop-signal task: Possible relevance to ADHD. Psychopharmacol. (Berl.).

[49] Benn A, Robinson ESJ (2017). Differential roles for cortical versus sub-cortical noradrenaline and modulation of impulsivity in the rat. Psychopharmacol. (Berl.).

[50] Steinmetz NA, Zatka-Haas P, Carandini M, Harris KD (2019). Distributed coding of choice, action and engagement across the mouse brain. Nature.

[51] Trivedi, P., Yu, H., MacNeil, D. J., Van Der Ploeg, L. H. T. & Guan, X. M. Distribution of orexin receptor mRNA in the rat brain. *FEBS Lett*. 10.1016/S0014-5793(98)01266-6 (1998). 10.1016/s0014-5793(98)01266-6 9821961

[52] Bourgin, P. et al. Hypocretin-1 modulates rapid eye movement sleep through activation of locus coeruleus neurons. *J. Neurosci*. **20**, 7760–7765 (2000). 10.1523/JNEUROSCI.20-20-07760.2000 PMC6772862 11027239

[53] Perugi G, Vannucchi G (2015). The use of stimulants and atomoxetine in adults with comorbid ADHD and bipolar disorder. Expert Opin. Pharmacother..

[54] Moy SS (2013). Disruption of social approach by MK-801, amphetamine, and fluoxetine in adolescent C57BL/6J mice. Neurotoxicol. Teratol..

[55] Eban-Rothschild, A., Rothschild, G., Giardino, W. J., Jones, J. R. & De Lecea, L. VTA dopaminergic neurons regulate ethologically relevant sleep-wake behaviors. *Nat. Neurosci*. **19**, 1356–1366 (2016). 10.1038/nn.4377 PMC5519826 27595385

[56] Li, S. Bin, Nevárez, N., Giardino, W. J. & De Lecea, L. Optical probing of orexin/hypocretin receptor antagonists. *Sleep* **41**, zsy141 (2018). 10.1093/sleep/zsy141 PMC6454482 30060151

[57] Adamantidis AR, Zhang F, Aravanis AM, Deisseroth K, De Lecea L (2007). Neural substrates of awakening probed with optogenetic control of hypocretin neurons. Nature.

[58] Carter ME, Borg JS, de Lecea L (2009). The brain hypocretins and their receptors: mediators of allostatic arousal. Curr. Opin. Pharmacol..

