# Supplementary material for: Optogenetic and pharmacological interventions link hypocretin neurons to impulsivity in mice
Source: Commun Biol. 2023 Jan 19;6:74. doi: 10.1038/s42003-023-04409-w (PMC9852239; doi:10.1038/s42003-023-04409-w)
Supplement: Supplementary file 1 — Supplementary Information [file 42003_2023_4409_MOESM1_ESM.pdf]

Supplementary Information

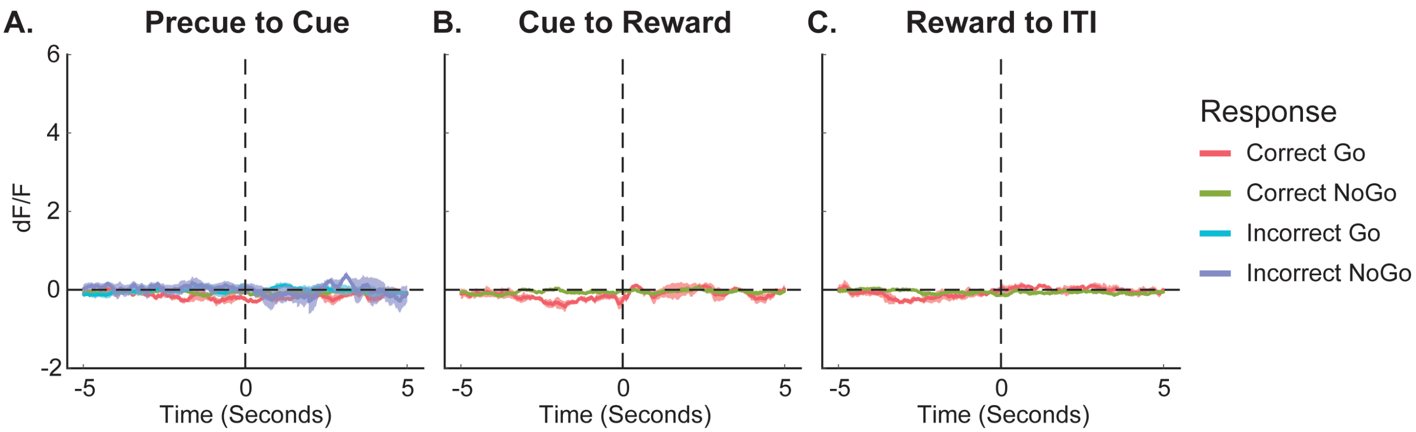

Supplementary Figure 1. Fiber Photometry in Control Mice. N=3 Wild-type (hcrt-cre-) mice were virus-treated, implanted, and recorded as described for experimental mice. Figure displays mean ΔF/F recorded 5 s before and 5 s after (A) Precue to Cue, (B) Cue to Reward, or (C) Reward to ITI transitions in the Go/NoGo task. Time 0 and vertical dotted line denote the transition point. Color hue denotes animals' response in the Go/NoGo task). Shaded region represents S.E.M. Bottom row. No control animal displayed consistent signal change at any point during Go/NoGo task.

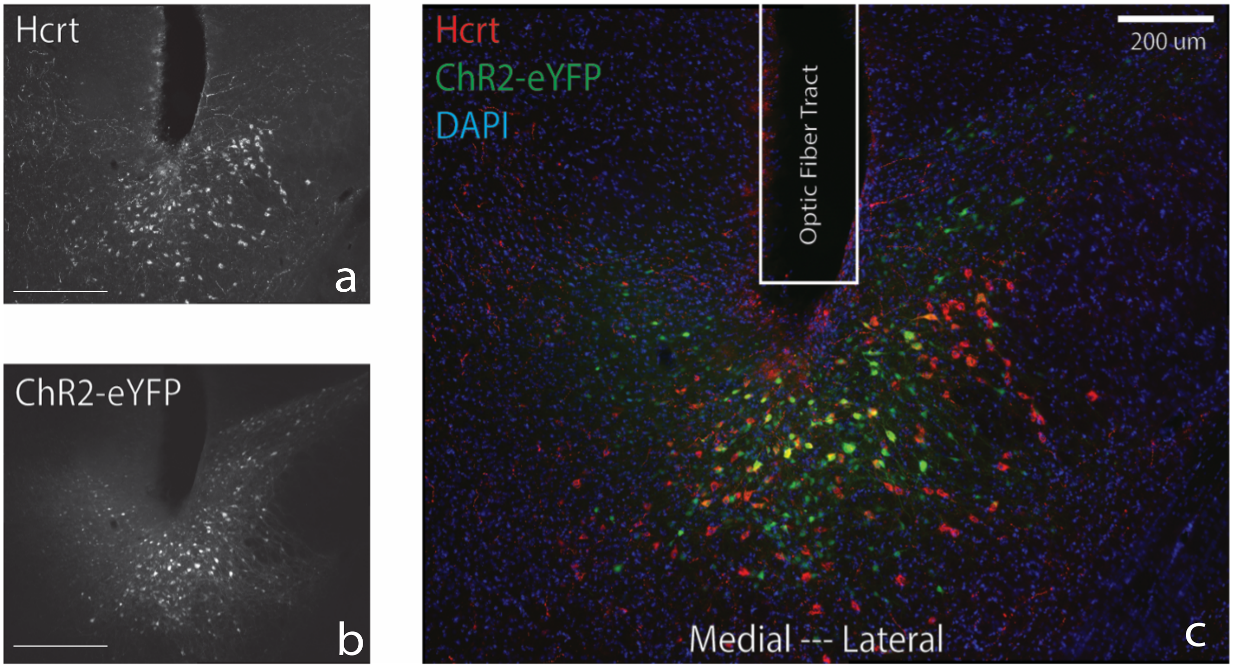

Supplementary Figure 2. AAV-DJ-ef1α-DIO-ChR2-eYFP expression in Hcrt-IRES-cre mice. Adult male Hcrt-IRES-cre mouse was perfused and brain tissue processed for immunofluorescence.. The brain was harvested two weeks after virus injection. Brain was sectioned at 40µm on a cryostat (Leica Microsystems) and hcrt expression visualized immunohistochemically as described in Methods using the following antibodies: goat anti-orexin A (1:1,000, Santa Cruz sc-8070) and donkey anti-goat Alexa Fluor 594 (1:500, Jackson ImmunoResearch Laboratories, Inc, 705-585-147). Tissue was mounted on gelatin coated slides (FD Neurotechnologies, Inc.; PO101) with Fluoroshield-containing DAPI Mounting Media (Sigma; F6057) and imaged on a confocal microscope (Zeiss LSM710) using ZEN software and minimally processed with ImageJ (NIH) to enhance brightness and contrast. a – Immunohistochemistry to Hcrt; b – immunofluorescence corresponding to the ChR2-expressing virus.Scale bars in a and b= 150 um c- Colocalization of Hcrt and ChR2 in superposed channels. More than 70% of eYFP labeled neurons were also positive for Hcrt (72 +13% n=487 cells).

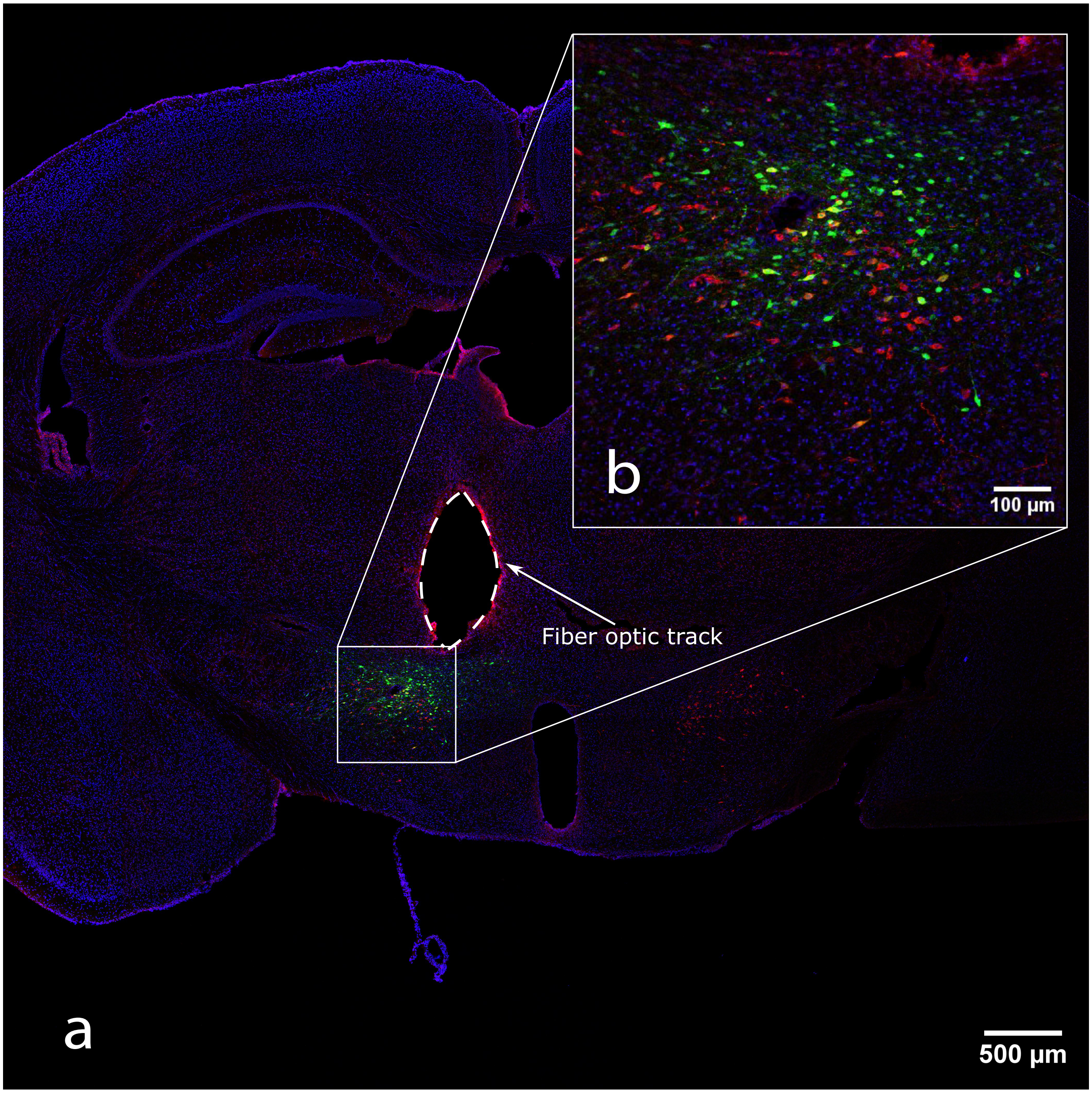

Supplementary Figure 3. **AAV-DJ-ef1α-DIO-GCamp6-eYFP expression in Hcrt-IRES-cre mice**

a)Immunofluorescence of the injection site of 0.3 ul AAV-DJ-DIO Gcamp6 virus in Hcrt-IRES cre mice. b) shows higher magnification of the boxed area double labeled with a goat Hcrt-1 antiserum (SantaCruz sc-8070) and donkey anti-goat Alexa Fluor 594. % GCamp positive cells GCaMP+ that were immunopositive = 52.76% +/- 26.29%%. Local injections reached to

approximately 50% of all Hcrt positive neurons as estimated by double labeling with GCamp (n=4 animals).
