# Supplementary material for: Optogenetic and pharmacological interventions link hypocretin neurons to impulsivity in mice
Source: Commun Biol. 2023 Jan 19;6:74. doi: 10.1038/s42003-023-04409-w (PMC9852239; doi:10.1038/s42003-023-04409-w)
Supplement: Supplementary file 2 — Description of Additional Supplementary Files [file 42003_2023_4409_MOESM2_ESM.pdf]

Description of Additional Supplementary Files

File name: Supplementary Movie 1
Description: Trial14-CorrNoGo nolaser. Represents a control NoGo session, where a cue is delivered during 10 seconds and the animal is expected to withhold the response during this time. Successful completion of the NoGo session delivers a reward and turns off the and cage light.

File name: Supplementary Movie 2
Description: Trial 4-IncorrNoGo-nogocuelaser. A No Go session where a laser delivering optogenetic stimulation to Hcrt neurons was triggered at the same time as the NoGo cue, showing that it elicited a premature response

File name: Supplementary Data 1
Description: Raw data source for Figures 3-5

File name: Supplementary Data 2
Description: Statistical Analyses.
