# Supplementary material for: Optogenetic and pharmacological interventions link hypocretin neurons to impulsivity in mice
Source: Commun Biol. 2023 Jan 19;6:74. doi: 10.1038/s42003-023-04409-w (PMC9852239; doi:10.1038/s42003-023-04409-w)
Supplement: Supplementary file 7 — Reporting Summary [file 42003_2023_4409_MOESM7_ESM.pdf]

nature research

Corresponding author(s): de Lecea, Luis

Last updated by author(s): Jan 21, 2022

Reporting Summary

Nature Research wishes to improve the reproducibility of the work that we publish. This form provides structure for consistency and transparency in reporting. For further information on Nature Research policies, see our Editorial Policies and the Editorial Policy Checklist.

Statistics

For all statistical analyses, confirm that the following items are present in the figure legend, table legend, main text, or Methods section.

n/a	Confirmed	
☐	☒	The exact sample size (*n*) for each experimental group/condition, given as a discrete number and unit of measurement
☐	☒	A statement on whether measurements were taken from distinct samples or whether the same sample was measured repeatedly
☐	☒	The statistical test(s) used AND whether they are one- or two-sided<br>*Only common tests should be described solely by name; describe more complex techniques in the Methods section.*
☐	☒	A description of all covariates tested
☐	☒	A description of any assumptions or corrections, such as tests of normality and adjustment for multiple comparisons
☐	☒	A full description of the statistical parameters including central tendency (e.g. means) or other basic estimates (e.g. regression coefficient) AND variation (e.g. standard deviation) or associated estimates of uncertainty (e.g. confidence intervals)
☐	☒	For null hypothesis testing, the test statistic (e.g. *F*, *t*, *r*) with confidence intervals, effect sizes, degrees of freedom and *P* value noted<br>*Give P values as exact values whenever suitable.*
☒	☐	For Bayesian analysis, information on the choice of priors and Markov chain Monte Carlo settings
☒	☐	For hierarchical and complex designs, identification of the appropriate level for tests and full reporting of outcomes
☒	☐	Estimates of effect sizes (e.g. Cohen's *d*, Pearson's *r*), indicating how they were calculated

Our web collection on statistics for biologists contains articles on many of the points above.

Software and code

Policy information about availability of computer code

Data collection	We used commercial software from Med Associates for data collection
Data analysis	Matlab code for data analysis will be made available upon request

For manuscripts utilizing custom algorithms or software that are central to the research but not yet described in published literature, software must be made available to editors and reviewers. We strongly encourage code deposition in a community repository (e.g. GitHub). See the Nature Research guidelines for submitting code & software for further information.

Data

Policy information about availability of data

All manuscripts must include a data availability statement. This statement should provide the following information, where applicable:

- Accession codes, unique identifiers, or web links for publicly available datasets
- A list of figures that have associated raw data
- A description of any restrictions on data availability

The datasets generated during and/or analysed during the current study are available from the corresponding author on reasonable request

April 2020

Field-specific reporting

Please select the one below that is the best fit for your research. If you are not sure, read the appropriate sections before making your selection.

☒ Life sciences ☐ Behavioural & social sciences ☐ Ecological, evolutionary & environmental sciences

For a reference copy of the document with all sections, see nature.com/documents/nr-reporting-summary-flat.pdf

Life sciences study design

All studies must disclose on these points even when the disclosure is negative.

Sample size	Group size was determined to be >6 animals by power analysis assuming a variation of ~20 % in the number of nosepokes between individuals.
Data exclusions	Only datapoints from animals whose viral injection was not on target were excluded.
Replication	All experiments were reproduced in different animals or cohorts of animals and the results were compared using appropriate statistical tests
Randomization	All animals received all treatments in a randomized manner
Blinding	A single experimenter collected all data from animals that were coded and their genotype revealed for analysis.

Reporting for specific materials, systems and methods

We require information from authors about some types of materials, experimental systems and methods used in many studies. Here, indicate whether each material, system or method listed is relevant to your study. If you are not sure if a list item applies to your research, read the appropriate section before selecting a response.

Materials & experimental systems

n/a	Involved in the study
☐	☒ Antibodies
☒	☐ Eukaryotic cell lines
☒	☐ Palaeontology and archaeology
☐	☒ Animals and other organisms
☒	☐ Human research participants
☒	☐ Clinical data
☒	☐ Dual use research of concern

Methods

n/a	Involved in the study
☒	☐ ChIP-seq
☒	☐ Flow cytometry
☒	☐ MRI-based neuroimaging

Antibodies

Antibodies used	We used commercial antisera to detect Hcrt
Validation	Antisera to Hcrt have been validated by immunoabsorption and lack of signal in mutant mice

Animals and other organisms

Policy information about studies involving animals; ARRIVE guidelines recommended for reporting animal research

Laboratory animals	Hrt IRES cre mice backcrossed for 11 generations or more into C57BL6 were used in this study maintained under controlled light dark cycle and constant conditions of humidity and temperature.
Wild animals	No wild animals were used in this study
Field-collected samples	No samples were collected in the field
Ethics oversight	All experiments were carried out in accordance with the US National Institutes of Health Guide for the Care and Use of Laboratory Animals guidelines and were approved by the Stanford University Administrative Panel on Laboratory Animal Care (protocol ID #18787).

Note that full information on the approval of the study protocol must also be provided in the manuscript.
